# Infant Methemoglobinemia: Fewtrell Responds

**Published:** 2005-12

**Authors:** Lorna Fewtrell

**Affiliations:** Centre for Research into Environment and Health, Crewe, Cheshire, United Kingdom, E-mail: lorna@creh.demon.co.uk

In her letter, Zeman seems to be objecting to three points relating my article (Fewtrell 2004): that the role of cofactors is not new, that her articles were not cited, and that exposure–response data are available.

First, in my article (Fewtrell 2004) I did not suggest that the role of cofactors was a novel discovery, as evidenced by the selection of articles I cited noting such factors. Rather, I noted the fact that the role of cofactors often seems to be overlooked in some of the literature.

Second, as stated in the conclusion (Fewtrell 2004), “the study did not set out to review the role of nitrates in the causation of methemoglobinemia” nor, by extension, the role of cofactors; thus the literature citation was selective.

Finally, I assessed the article by Zeman et al. (2002) in the literature review for my study (Fewtrell 2004), but I felt it did not provide useful drinking water (i.e., exposure) data related to the level of methemoglobinemia in infants (i.e., response data); therefore, the article by Zeman et al. (2002) was not cited (although I do consider the new data presented in Zeman’s letter to be of interest).

Three points that influenced my decision not to cite the article by Zeman et al. (2002) are worth noting. First, Zeman et al.’s Figure 2 shows an apparent relationship between nitrate level in wells (parts per million) and “nitrate” (this is presumably nitrite, as described in the figure legend and Zeman’s letter) exposure in milligrams per kilogram per day. This was reported to have a correlation of 0.71, presumably resulting in a coefficient of determination of 0.50. However, some reported concentrations are remarkably high, exceeding 1,000 ppm nitrate. The reported relationship appears, visually, to be dependent on a few very high value(s) for the claimed correlation, making inference—or application of the functional relationship—within more usual exposure levels inappropriate. The data points within more “usual” elevated exposure ranges (say < 500 ppm) do not appear to exhibit a clear correlation between nitrate concentration (parts per million) in well water and calculated nitrite intake in milligrams per kilogram per day.

Second, this figure simply claims a correlation between a concentration (i.e., nitrate in water, parts per million) and a precurser of the outcome condition (i.e., calculated nitrite intake, milligrams per kilogram per day), not the “outcome” of interest I discussed (Fewtrell 2004). It is unclear how the boiling of water (which may lead to an increase in nitrate concentrations) is accounted for in the relationship presented by Zeman et al. (2002). The relationship is also likely to be location specific, being dependent upon local feeding habits [e.g., level of formula, tea (chi), vegetables, etc., given to the infant].

Third, Zeman et al. (2002) did not provide a detailed explanation of how the dependent variable numerical values in their Figure 2 were derived, making it difficult to assess the quality of this information.

The selection of appropriate studies to include in any global assessment is difficult and will always be contentious. I hope that these observations explain my decision not to cite the article by Zeman et al. (2002).
